# Advances of surface-enhanced Raman and IR spectroscopies: from nano/microstructures to macro-optical design

**DOI:** 10.1038/s41377-021-00599-2

**Published:** 2021-08-04

**Authors:** Hai-Long Wang, En-Ming You, Rajapandiyan Panneerselvam, Song-Yuan Ding, Zhong-Qun Tian

**Affiliations:** 1 State Key Laboratory of Physical Chemistry of Solid Surfaces, Collaborative Innovation Center of Chemistry for Energy Materials, College of Chemistry and Chemical Engineering, Xiamen University, Xiamen, 361005 China; 2 Department of Chemistry, SRM University- AP, Amaravathi, Andhra Pradesh India

**Keywords:** Optics and photonics, Optical physics

## Abstract

Raman and infrared (IR) spectroscopy are powerful analytical techniques, but have intrinsically low detection sensitivity. There have been three major steps (i) to advance the optical system of the light excitation, collection, and detection since 1920s, (ii) to utilize nanostructure-based surface-enhanced Raman scattering (SERS) and surface-enhanced infrared absorption (SEIRA) since 1990s, and (iii) to rationally couple (i) and (ii) for maximizing the total detection sensitivity since 2010s. After surveying the history of SERS and SEIRA, we outline the principle of plasmonics and the different mechanisms of SERS and SEIRA. We describe various interactions of light with nano/microstructures, localized surface plasmon, surface plasmon polariton, and lightning-rod effect. Their coupling effects can significantly increase the surface sensitivity by designing nanoparticle–nanoparticle and nanoparticle–substrate configuration. As the nano/microstructures have specific optical near-field and far-field behaviors, we focus on how to systematically design the macro-optical systems to maximize the excitation efficiency and detection sensitivity. We enumerate the key optical designs in particular ATR-based operation modes of directional excitation and emission from visible to IR spectral region. We also present some latest advancements on scanning-probe microscopy-based nanoscale spectroscopy. Finally, prospects and further developments of this field are given with emphasis on emerging techniques and methodologies.

## Abstract (web summary)

This review focuses on the advance of optical design from nano/micro to macro in SERS and SEIRA, especially on the optical coupling between nano/micro and macro scales.

## Graphical abstract

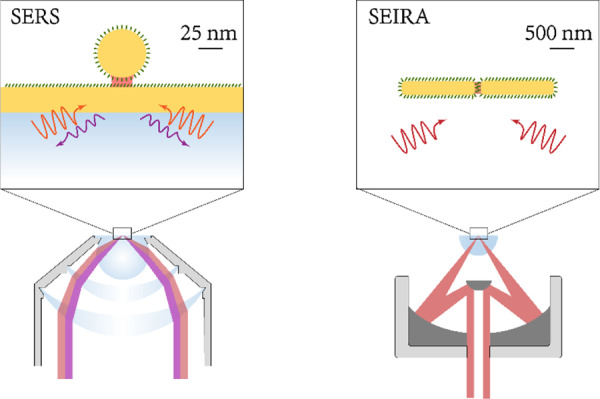

## Introduction

Raman scattering spectroscopy and infrared absorption spectroscopy are two important spectroscopic techniques that can provide molecular or lattice vibrational fingerprint information^1^. Their ability for chemical identification triggered many scientists to develop infrared spectroscopy for surface analysis in the 1960s^2^ and Raman spectroscopy at surfaces and interfaces in the 1970s^3–5^. However, it was challenging to directly detect monolayer or even sub-monolayer molecules due to low detection sensitivity. Thus, for a long time, Raman and infrared spectroscopy were applied for characterizing structures of bulk solid or liquid samples.

In 1974, Fleischmann et al. reported unprecedentedly intense surface Raman spectra of pyridine molecules adsorbed on electrochemically roughened silver electrodes^3^. After reading their paper, Van Duyne et al. undertook their experiments then made the calculation carefully. In 1977 they reported the surface-enhanced Raman scattering (SERS) effect that the Raman intensities of adsorbates on the electrodes could be boosted up to a million-fold^5^. In 1978, Moskovits attributed this strong Raman enhancement to the resonant excitation of surface plasmons (SPs) at the roughened Ag electrode surfaces and predicted that similar phenomena could also be observed in Ag and Cu colloids^6^. In 1979, Creighton and coworkers observed the giant Raman scattering effect on Ag and Au colloids (as called nanoparticle aggregates now)^7^. In 1980, the first observation of the surface-enhanced infrared absorption spectroscopy (SEIRA) was reported by Hartstein et al. from molecular monolayers due to thin metal overlayers and underlayers in the attenuated-total-reflection (ATR) geometry^8^. The total enhancement including contributions from the ATR geometry was almost 10^4^. They attributed the IR enhancement to an electric field enhancement due to the resonant excitation of SPs with the island nature of the thin metal films.

In the 1990s, SERS and SEIRA attracted a wide interest again by the rapid developments in nanoscience^9–16^. Many relevant techniques have been employed to controllably synthesize and characterize SERS-related nanoparticles or nanostructures. It was found that the SERS activity was critically dependent on the size, shape, nature, morphology, and nanometer-sized gaps (nanogaps) in/between nanoparticles^17^. Such efforts have led to the most significant progress in this field. High-quality SERS spectra from a single molecule adsorbed on well-characterized nanoparticles have been obtained by Nie and Kneipp groups respectively in 1997^14,15^. Xu et al. further demonstrated that single-molecule SERS worked in an Ag nanoparticle dimer or oligomers with a nanogap^16^. Now, SERS is considered as a phenomenon associated with the amplification by several orders of magnitude of Raman signals of analyte located at or very close to metallic nanostructures which support hotspots (nanoscale regions with a strongly enhanced local electromagnetic (EM) field) mainly due to the excitation of SPs^17,18^. Some examples of SERS-active nanostructures are Au and Ag nanoparticles with nanogaps and nanometer-sized tips (nanotips), and structured surfaces with nanometer-sized holes (nanoholes), voids, bumps, grooves, or ridges^17^. Typical SERS substrates like metal nanostructures^19^, metal-organic frameworks (MOF)-based substrates^20^, general core–shell substrates^21,22^, and metal-coated pillar substrates^23,24^, etc. have been fabricated and realized high sensitivity and selectivity of sample materials from solid, liquid even to gas.

The SERS-active nanostructures are usually called plasmonic optical antennas that not only act as receiving antenna for local field enhancement of incident light at hotspots but also act as transmitting antenna for transmitting the local signals from molecular Raman emitters located at hotspots to far-field where the detector is located. Two major factors should be carefully considered: (1) The excitation optics including wavelength, incident angles, polarization states, beam shapes, etc., determines the coupling efficiency between the incident light and SERS-active nanostructures, which directly defines the local EM field enhancement at hotspots; (2) the collection optics determines how much Raman-scattered EM field generated by the molecules nearby the nanostructure could be transmitted to the detector by means of the molecular emitter-nanostructure coupling since SERS-active nanostructures efficiently and directionally emit Raman signals. In summary, the substrate’s materials and nanostructures, and excitation/collection optics are the key elements determining the detection sensitivity of SERS.

**Fig. 1:**
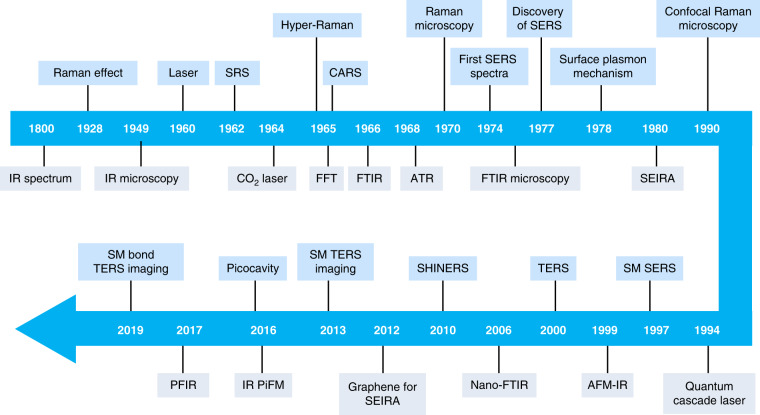
A timeline of historical advances in SERS, SEIRA, and related techniques. SRS stimulated Raman scattering, Hyper-Raman Hyper-Raman scattering, CARS coherent anti-Stokes Raman scattering, FFT fast Fourier transform, FTIR Fourier transform infrared spectroscopy, ATR Attenuated total reflectance, SEIRA surface-enhanced infrared absorption spectroscopy, SM single molecule, TERS tip-enhanced Raman spectroscopy, SHINERS shell-isolated nanoparticle-enhanced Raman spectroscopy, PFIR, peak force infrared microscopy, IR PiFM, infrared photoinduced force microscopy, Nano-FTIR Fourier transform infrared nanospectroscopy, AFM-IR Atomic force microscopy-based infrared spectroscopy

However, traditional SERS substrates suffer from the severe limitation on spatial resolution and interference between the sample molecules and SERS substrates^25,26^. Several other Raman spectroscopies have been developed, including tip-enhanced Raman spectroscopy (TERS)^27–30^, shell-isolated nanoparticle-enhanced Raman spectroscopy (SHINERS)^31^, and surface-enhanced nonlinear spectroscopies such as hyper-Raman spectroscopy^32^, femtosecond stimulated Raman spectroscopy^33^. Collectively, the above-mentioned techniques are often referred as plasmon-enhanced Raman spectroscopy (PERS)^18^, whose timeline of milestone developments is summarized in Fig. 1.

Parallelly, several scientists tried to improve the sensitivity of other spectroscopic techniques beyond SERS in the 1980s. Similar to SERS, local EM enhancement is the dominant contribution to SEIRA. While most SEIRA-active substrates were metallic nano/microstructures in the early days^34^, III–V semiconductors, voltage-tuned graphene nanoribbons, and nanodiscs, and hexagonal boron nitride, etc., have been adopted as plasmonic materials for the mid-infrared region more recently^35–39^.

Infrared spectroscopy with ultrahigh spatial resolution represents one of the important variants of SEIRA. In 1985, infrared spectroscopy with the sub-wavelength spatial resolution was developed based on an aperture-scanning near-field optical microscope^40^. In 1999, nanoscale infrared spectroscopy based on scattering-type- (i.e., apertureless-) scanning near-field optical microscope (s-SNOM) was developed^41^. In s-SNOM, the nanoscale IR signals can also be moderately enhanced by a sharp metallic tip. See the timeline of milestone developments of SEIRA and nanoscale IR spectroscopy in Fig. 1. Up to now, the sensitivity of SEIRA only reaches hundreds of oscillators, and cannot reach a single oscillator. Actually, the development of SEIRA-active substrates’ materials, nanostructures, and excitation/collection optics lags far behind the developments in SERS.

**Fig. 2:**
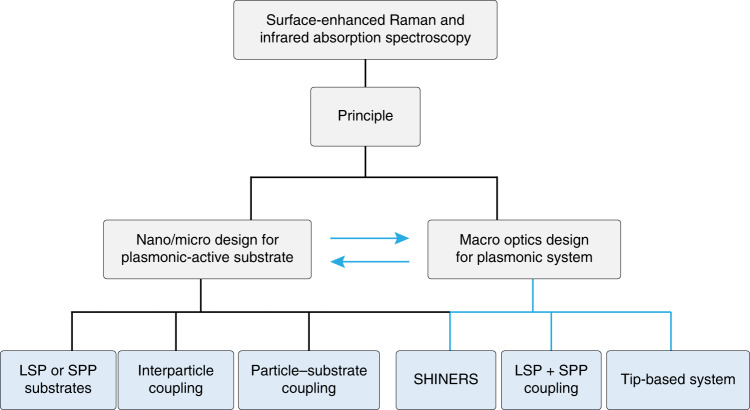
Optical design from nano/micro substrates to macro-optics for SERS and SEIRA. Nano/micro designs are the key elements of SERS and SEIRA, which determines the sensitivity of SERS and SEIRA. Macro-optical designs depend on the features of nano/micro structured substrates

In this review, we first cover the EM theories of SERS and SEIRA with emphasis on the local EM field enhancement due to the excitation of localized surface plasmon (LSP), surface plasmon polariton (SPP), lightning-rod effect (LRE), and particularly, the coupling effect among them. We then sketch the developments of coupled nanostructures to significantly increase the detection sensitivity in nanoparticle–nanoparticle, and nanoparticle–substrate configuration. After that, we focus on how to systematically design the macro excitation/collection optical systems capable of fitting the specific nano/microstructures to maximize the local field and radiation field enhancement for ultrasensitive SERS and SEIRA measurements. Finally, we discuss and assess some new designs for SERS and SEIRA techniques. The overall logical framework is shown in Fig. 2.

## Principles of SERS and SEIRA

### Electromagnetic theories of SERS and SEIRA

The following equation gives the Raman intensity expression of a surface-enhanced Raman spectrum for a vibrational mode of a molecule following the Placzek’s polarizability theory with regard to the instrumental and surface factors^42^,

1 $$I_{{{{\mathrm{SEIRA}}}}}^{{{\mathrm{k}}}} = \frac{{2^7}}{{3^2}}\frac{{\pi ^5}}{{{{{\mathrm{c}}}}^4}}I_0\left( {\upsilon _0 - \upsilon _{{{{\mathrm{k,mn}}}}}} \right)^4\mathop {\sum}\limits_{\rho \sigma } {\left| {\left( {\alpha _{\rho \sigma }} \right)_{{{{\mathrm{mn}}}}}} \right|^2} NA{\Omega}QT_{{{\mathrm{m}}}}T_0 \cdot G_{{{{\mathrm{SERS}}}}}$$

The SEIRA intensity follows a similar expression:

2 $$I_{{{{\mathrm{SEIRA}}}}}^k \propto K\left| {\frac{{d{{{\mathbf{\mu }}}}}}{{dQ_k}} \cdot {{{\boldsymbol{E}}}}_{{{\mathrm{0}}}}} \right|^2NA{\Omega}QT_mT_0 \cdot G_{{{{\mathrm{SEIRA}}}}}$$

where *I*_0_ is the incident intensity, *υ*_0_ and *υ*_k,mn_ are the frequencies (cm^−1^) of the incident light and the *k*^th^ vibrational normal mode, respectively. *N* is the number density of the adsorbates on the substrate (molecules cm^−2^). *A* is the surface area illuminated by the laser beam (cm^2^). *Ω* is the solid angle of the collection optics (sr). *QT*_m_*T*_0_ is the product of the detector efficiency, the throughput of the dispersion system and the transmittance of the collection optics. (*α*_*ρσ*_)_mn_ is the *ρσ* component of the adsorbate’s polarizability derivative with respect to the *k*th normal mode. d***μ***/d*Q*_k_ is the adsorbate’s electric dipole derivative with respect to the *k*^th^ normal mode. Even if the probe molecules fully cover a flat surface, the number of molecules is only about 10^7^ within the laser spot of about 1 μm in diameter in SERS. Considering that typically one Raman photon is produced by about 10^10^ incident photons, it is still not sufficient to detect a surface adsorbate with a monolayer coverage nowadays even with the state-of-the-art Raman instruments through the improvement on *QT*_m_*T*_0_. Usually, researchers do not increase the incident power to prevent surface/analyte damage, but the enhancement factor *G*_SERS_ and *G*_SEIRA_.

**Fig. 3:**
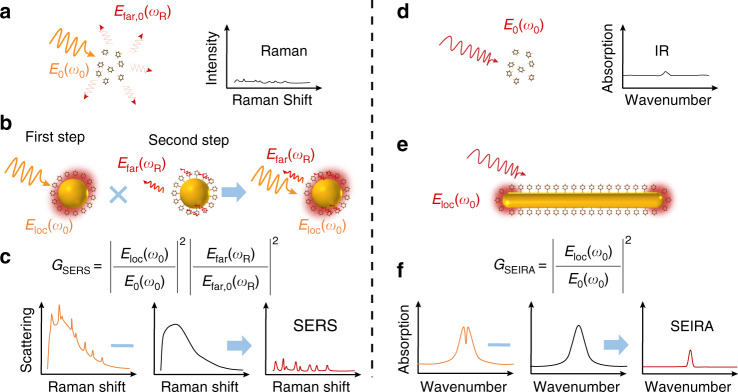
Principles of SERS and SEIRA. **a** Normal Raman scattering $E_{\rm{far,0}}\left( {\omega _{\rm{R}}} \right)$ and Raman spectrum of molecules illuminated by narrow band laser. **b** Surface-enhanced Raman scattering from molecules adsorbed onto the metal nanosphere. Local field $E_{\rm{loc}}\left( {\omega _0} \right)$ around metal nanosphere is enhanced by localized surface polasmon polaritons (LSP) around the metal nanosphere. The excitation and radiation efficiency $E_{{{{\mathrm{far}}}}}\left( {\omega _{{{\mathrm{R}}}}} \right)$ of Raman scattering from molecules are improved in the local field via the interaction with LSP. Thus, the Raman scattering will be enhanced by $G_{\rm{SERS}}$**. c** Data processing for SERS spectrum. Raw spectrum (left) from molecules adsorbed on nanosphere contains photoluminescence (PL) spectrum (middle) and molecular SERS spectrum (right). **d** Normal IR spectrum of molecules illuminated by IR laser. **e** LSP around metal nanorod is excited by IR laser. Local field $E_{\rm{loc}}\left( {\omega _0} \right)$ at the nanorod’s two ends is enhanced by LSP. IR absorption of molecules in the local field is enhanced by $G_{\rm{SEIRA}}$. **f** Data processing for SEIRA. Raw spectrum (left) from molecules adsorbed on nanorod contains nanorod (middle) and molecular absorption (right). Owing to the coupling between the plasmon and the molecular vibration, the SEIRA spectrum (left) often shows a asymmetric peak or a dip when the molecules absorb IR laser

In order to know how to improve *G*_SERS_ and *G*_SEIRA_, it is necessary to understand the process of SERS and SEIRA. In SERS as shown in Fig. 3a, b, the induced dipole ${{{\boldsymbol{p}}}}_m\left( {\omega _R,{{{\boldsymbol{r}}}}_m} \right)$ at the Raman scattering frequency (ω_R_), can be approximately expressed with the inner product of two independent quantities (Eq. 3)^21^,

3 $${{{\boldsymbol{p}}}}_{\rm{m}}\left( {\omega _{\rm{R}},{{{\boldsymbol{r}}}}_{\rm{m}}} \right) = {{{\mathbf{\alpha }}}}_{\rm{m}}^{\rm{I}}\left( {\omega _{\rm{R}},\omega _0} \right) \cdot {{{\boldsymbol{E}}}}_{\rm{loc}}\left( {\omega _0,{{{\boldsymbol{r}}}}_{\rm{m}}} \right)$$

4 $${{{\boldsymbol{E}}}}_{\rm{loc}}\left( {\omega _0,{{{\boldsymbol{r}}}}_{\rm{m}}} \right) = g_1\left( {\omega _0,{{{\boldsymbol{r}}}}_{\rm{m}}} \right){{{\boldsymbol{E}}}}_0\left( {\omega _0} \right)$$

where ***E***_loc_ and ***E***_0_ are the local and incident electric field strength at the position ***r***_m_ where the molecules are located in the presence and absence of an optical antenna at the incident frequency *ω*_0_, respectively. *g*_1_(*ω*_0_, ***r***_m_) is the enhancement factor of the incident electric field strength. ${{{\mathbf{\alpha }}}}_{{{\mathrm{m}}}}^{{{\mathrm{I}}}}\left( {\omega _R,\omega _0} \right)$ is the Raman polarizability derivatives at Raman scattering frequency *ω*_R_ under the illumination of an incident laser at the incident frequency *ω*_0_. Furthermore, the antenna at ***r***_A_ would also be locally excited by the Raman-scattered dipolar source ***p***_m_ (*ω*_R_, ***r***_m_) nearby. Then in the induced dipole approximation, the induced dipole of the antenna ***p***_A_ (*ω*_R_, ***r***_A_) could be expressed by,

5 $${{{\boldsymbol{p}}}}_{{{\mathrm{A}}}}\left( {\omega _{{{\mathrm{R}}}},{{{\boldsymbol{r}}}}_{{{\mathrm{A}}}}} \right) = \omega _{{{\mathrm{R}}}}^2\mu \mu _0{{{\mathbf{\alpha }}}}_{{{\mathrm{A}}}}\left( {\omega _{{{\mathrm{R}}}},{{{\boldsymbol{R}}}}} \right) \cdot {{{\boldsymbol{G}}}}_{{{{\mathrm{A}}}} - {{{\mathrm{m}}}}}\left( {{{{\boldsymbol{R}}}},{{{\boldsymbol{r}}}}_{{{\mathrm{m}}}}} \right) \cdot {{{\boldsymbol{p}}}}_{{{\mathrm{m}}}}\left( {\omega _{{{\mathrm{R}}}},{{{\boldsymbol{r}}}}_{{{\mathrm{m}}}}} \right)$$

where ${{{\mathbf{\alpha }}}}_{{{\mathrm{A}}}}\left( {\omega _{{{\mathrm{R}}}},{{{\boldsymbol{R}}}}} \right)$ is the polarizability of the antenna. ${{{\boldsymbol{G}}}}_{{{{\mathrm{A}}}} - {{{\mathrm{m}}}}}\left( {{{{\boldsymbol{R}}}},{{{\boldsymbol{r}}}}_{{{\mathrm{m}}}}} \right)$ is the dyadic Green’s function. The total signals detected by the detector at far-field, come from the additive local source ***p***(*ω*_R_),

6 $${{{\boldsymbol{p}}}}\left( {\omega _{{{\mathrm{R}}}}} \right) = {{{\boldsymbol{p}}}}_{{{\mathrm{m}}}}\left( {\omega _{{{\mathrm{R}}}},{{{\boldsymbol{r}}}}_{{{\mathrm{m}}}}} \right) + {{{\boldsymbol{p}}}}_{{{\mathrm{A}}}}\left( {\omega _{{{\mathrm{R}}}},{{{\boldsymbol{r}}}}_{{{\mathrm{A}}}}} \right)$$

7 $${{{\boldsymbol{p}}}}\left( {\omega _{{{\mathrm{R}}}}} \right) = \left( {1 + \omega _{{{\mathrm{R}}}}^2\mu \mu _0{{{\mathbf{\alpha }}}}_{{{\mathrm{A}}}}\left( {\omega _{{{\mathrm{R}}}},{{{\boldsymbol{R}}}}} \right) \cdot {{{\boldsymbol{G}}}}_{{{{\mathrm{A}}}} - {{{\mathrm{m}}}}}\left( {{{{\boldsymbol{R}}}},{{{\boldsymbol{r}}}}_{{{\mathrm{m}}}}} \right)} \right) \cdot {{{\boldsymbol{p}}}}_{{{\mathrm{m}}}}\left( {\omega _{{{\mathrm{R}}}},{{{\boldsymbol{r}}}}_{{{\mathrm{m}}}}} \right) = {{{\boldsymbol{p}}}}_{{{\mathrm{m}}}}\left( {\omega _{{{\mathrm{R}}}},{{{\boldsymbol{r}}}}_{{{\mathrm{m}}}}} \right)g_2\left( {\omega _{{{\mathrm{R}}}},{{{\boldsymbol{r}}}}_{{{\mathrm{A}}}}} \right)$$

Substitute Eqs. 3 and 4 into Eq. 7,

8 $${{{\boldsymbol{p}}}}\left( {\omega _{{{\mathrm{R}}}}} \right) = \left[ {g_1\left( {\omega _0,{{{\boldsymbol{r}}}}_{{{\mathrm{m}}}}} \right)} \right] \cdot \left[ {g_2\left( {\omega _{{{\mathrm{R}}}},{{{\boldsymbol{r}}}}_{{{\mathrm{A}}}}} \right){{{\mathbf{\alpha }}}}_{{{\mathrm{m}}}}^I\left( {\omega _{{{\mathrm{R}}}},\omega _0} \right) \cdot {{{\boldsymbol{E}}}}_0\left( {\omega _0} \right)} \right]$$

The total Raman intensity of SERS *I*_SERS_(*ω*_R_) is proportional to | ***p***(*ω*_R_) |^2^,

9 $$I_{{{{\mathrm{SERS}}}}}\left( {\omega _{{{\mathrm{R}}}}} \right) \propto \left| {g_1\left( {\omega _0,{{{\boldsymbol{r}}}}_{{{\mathrm{m}}}}} \right)} \right|^2 \cdot \left| {g_2\left( {\omega _{{{\mathrm{R}}}},{{{\boldsymbol{r}}}}_{{{\mathrm{A}}}}} \right){{{\mathbf{\alpha }}}}_{{{\mathrm{m}}}}^I\left( {\omega _{{{\mathrm{R}}}},\omega _0} \right) \cdot {{{\boldsymbol{E}}}}_0\left( {\omega _0} \right)} \right|^2$$

The total Raman intensity of normal Raman *I*_NR_(*ω*_R_) is,

10 $$I_{{{{\mathrm{NR}}}}}\left( {\omega _{{{\mathrm{R}}}}} \right) \propto \left| {{{{\mathbf{\alpha }}}}_{{{\mathrm{m}}}}^I\left( {\omega _{{{\mathrm{R}}}},\omega _0} \right) \cdot {{{\boldsymbol{E}}}}_0\left( {\omega _0} \right)} \right|^2$$

Thus, the SERS enhancement factor is^43,44^,

11 $$G_{{{{\mathrm{SERS}}}}} = \frac{{I_{{{{\mathrm{SERS}}}}}\left( {\omega _{{{\mathrm{R}}}}} \right)}}{{I_{{{{\mathrm{NR}}}}}\left( {\omega _{{{\mathrm{R}}}}} \right)}} = \left| {g_1\left( {\omega _0,{{{\boldsymbol{r}}}}_{{{\mathrm{m}}}}} \right)} \right|^2 \cdot \left| {g_2\left( {\omega _{{{\mathrm{R}}}},{{{\boldsymbol{r}}}}_{{{\mathrm{A}}}}} \right)} \right|^2$$

where

$$\left| {g_1\left( {\omega _0,{{{\boldsymbol{r}}}}_{{{\mathrm{m}}}}} \right)} \right|^2 = \left| {\frac{{{{{\boldsymbol{E}}}}_{{{{\mathrm{loc}}}}}\left( {\omega _0} \right)}}{{{{{\boldsymbol{E}}}}_0\left( {\omega _0} \right)}}} \right|^2,\;\left| {g_2\left( {\omega _{{{\mathrm{R}}}},{{{\boldsymbol{r}}}}_{{{\mathrm{A}}}}} \right)} \right|^2 = \left| {\frac{{{{{\boldsymbol{E}}}}_{{{{\mathrm{far}}}}}\left( {\omega _R} \right)}}{{{{{\boldsymbol{E}}}}_{{{{\mathrm{far}}}},0}\left( {\omega _R} \right)}}} \right|^2$$

The IR and SEIRA processes are shown in Fig. 3d, e. IR intensity is proportional to the normal of the inner product of two independent quantities, the electric dipole derivative with respect to the *k*th vibrational normal modes ${{{\mathbf{\mu }}}}_0\left( {\omega _k} \right)$ at the IR absorption frequency (ω_k_) and the local field around molecules ${{{\boldsymbol{E}}}}_0\left( {\omega _k} \right)$ (Eq. 12)

12 $$I_{{{{\mathrm{IR}}}}}\left( {\omega _{{{\mathrm{R}}}}} \right) \propto \left| {{{{\mathbf{\mu }}}}_0\left( {\omega _{{{\mathrm{k}}}}} \right) \cdot {{{\boldsymbol{E}}}}_0\left( {\omega _R} \right)} \right|^2.$$

The total IR intensity $I_{{{{\mathrm{SEIRA}}}}}\left( {\omega _k} \right)$ of SEIRA is proportional to $\left| {{{{\mathbf{\mu }}}}_0\left( {\omega _k} \right) \cdot E_{loc}\left( {\omega _k} \right)} \right|^2$,

13 $$I_{{{{\mathrm{SEIRA}}}}}\left( {\omega _{\rm{k}}} \right) \propto \left| {{{{\mathbf{\mu }}}}_0\left( {\omega _{\rm{k}}} \right) \cdot {{{\boldsymbol{E}}}}_{{{{\mathrm{loc}}}}}\left( {\omega _{\rm{k}}} \right)} \right|^2 = \left| {g_1\left( {\omega _{\rm{k}},{{{\boldsymbol{r}}}}_{{{\mathrm{m}}}}} \right)} \right|^2 \cdot \left| {{{{\mathbf{\mu }}}}_0\left( {\omega _{{{\mathrm{k}}}}} \right) \cdot {{{\boldsymbol{E}}}}_0\left( {\omega _{\rm{k}}} \right)} \right|^2$$

where the ***E***_loc_(*ω*_k_) is the local field enhanced by SEIRA structures,

14 $${{{\boldsymbol{E}}}}_{\rm{loc}}\left( {\omega _{\rm{k}},{{{\boldsymbol{r}}}}_{\rm{m}}} \right) = g_1\left( {\omega _{\rm{k}},{{{\boldsymbol{r}}}}_{\rm{m}}} \right){{{\boldsymbol{E}}}}_0\left( {\omega _{\rm{k}}} \right)$$

Thus, the SEIRA enhancement factor is

15 $$G_{{{{\mathrm{SEIRA}}}}} = \frac{{I_{{{{\mathrm{SEIRA}}}}}\left( {\omega _{\rm{k}}} \right)}}{{I_{{{{\mathrm{IR}}}}}\left( {\omega _{\rm{k}}} \right)}} = \left| {g_1\left( {\omega _{\rm{k}},{{{\boldsymbol{r}}}}_{{{\mathrm{m}}}}} \right)} \right|^2$$

In the EM theory of SERS (Fig. 3a, b and Eq. 11), two-step enhancement should be considered: (1) Local field enhancement of incident light $\left( {\left| {g_1\left( {\omega _0,{{{\boldsymbol{r}}}}_{{{\mathrm{m}}}}} \right)} \right|^2} \right)$ in the vicinity of the nanostructures at which the analytes are located at or very close to; (2) radiation field enhancement of the Raman-scattered light $\left( {\left| {g_2\left( {\omega _{{{\mathrm{R}}}},{{{\boldsymbol{r}}}}_{{{\mathrm{A}}}}} \right)} \right|^2} \right)$ due to the local excitation of the nanostructures by ***p***_m_(*ω*_R_, ***r***_m_) of the analytes. While in SEIRA (Fig. 3d-e and Eq. 15), the enhanced absorption intensity of the molecules is proportional to the local field enhancement of incident light $\left( {\left| {g_1\left( {\omega _0,{{{\boldsymbol{r}}}}_{{{\mathrm{m}}}}} \right)} \right|^2} \right)$. Several effects can result in the enhanced local EM field, such as surface plasmon resonance (SPR) especially the localized surface plasmon resonance (LSPR) of nanostructures and LRE in a metal nanotip^45^. In the next section, we will briefly discuss the effect of the plasmon-enhanced EM field.

### Plasmon-enhanced electromagnetic field for SERS and SEIRA

**Fig. 4:**
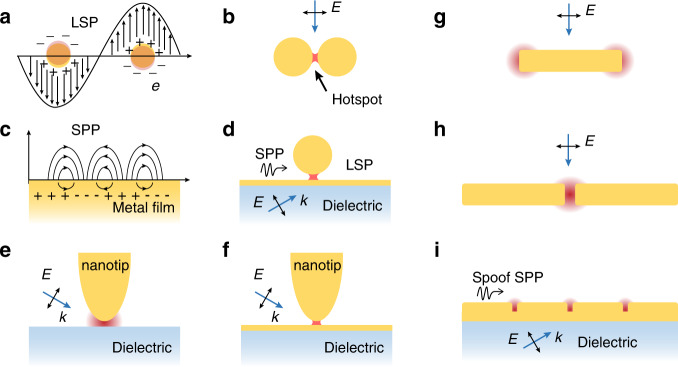
SERS and SEIRA-active nano/microstructures. **a** A single nanoparticle supporting a LSP, and **b** a nanoparticle dimer with a nanogap supporting a coupled LSP. **c** A metal film supporting a SPP, and **d** a particle-on-film coupled structure supporting the SPP-LSP coupling. **e** A nanotip supporting LSP and acting as a lightning rod, and **f** a nanotip-film coupled structure. **g** A nanorod, **h** A nanorod dimer. **i** A metal strip with periodic grooves supporting a SPP in IR region, which is called a spoof spp. **a**–**f** the substrates for SERS, **g**–**i** the substrates for SEIRA

The conduction electrons in metal or metal-like nanomaterials can be coherently excited by the incident light and collectively oscillate at the metal-dielectric interfaces^46^. Meanwhile, a resonance EM field is generated around the metal-dielectric interfaces. The collective oscillating electrons and the resonance EM field are called SPs as a whole. The dielectric constants and interfacial structure are two main factors to excite SPs. Materials that support SPs at a certain wavelength through structural modulation can be called as plasmonic materials such as Au, Ag, Cu in the visible and III–V semiconductors, and graphene in the mid-IR^47^. There are two types of SPs: (i) LSP (Fig. 4a), in which coherent electrons oscillate on the nanoparticle surfaces, and (ii) SPP (Fig. 4c), in which coherent electrons oscillate on the metal surfaces.

To understand the LSP and the local EM field enhancement of plasmonic nanostructures, we consider the ***E***_loc_ outside an Au nanosphere dispersed in bulk dielectric medium (with dielectric constant *ε*_d_), under the electrostatic approximation^46,48^,

16 $$E_{{{{\mathrm{loc}}}}}\left( {\omega _0,r_{{{\mathrm{m}}}}} \right) = E_0\left( {\omega _0} \right)\hat z - E_0\left( {\omega _0} \right)\frac{{\varepsilon _{{{\mathrm{m}}}} - \varepsilon _{{{\mathrm{d}}}}}}{{\varepsilon _{{{\mathrm{m}}}} + 2\varepsilon _{{{\mathrm{d}}}}}}\left[ {\frac{{\hat z}}{{r^3}} - \frac{{3z}}{{r^5}}{{{\boldsymbol{r}}}}_{{{\mathrm{m}}}}} \right]$$

The corresponding frequency-dependent extinction spectrum *I*_LSP_, i.e., LSPR spectrum could further be derived as^46,48^,

17 $$I_{{{{\mathrm{LSP}}}}}\left( {\omega _0} \right) = 12\,\pi \,{{{\mathrm{N}}}}a^3\varepsilon _{{{\mathrm{d}}}}^{3/2}\omega _0/{{{\mathrm{c}}}}\left[ {\frac{{{{{\mathrm{Im}}}}\left( {\varepsilon _{{{\mathrm{M}}}}\left( {\omega _0} \right)} \right)}}{{\left( {{{{\mathrm{Re}}}}(\varepsilon _{{{\mathrm{M}}}}(\omega _0)) + 2\varepsilon _{{{\mathrm{d}}}}} \right)^2 + ({{{\mathrm{Im}}}}(\varepsilon _{{{\mathrm{M}}}}(\omega _0)))^2}}} \right]$$

Equations 16 and 17 show that both ***E***_loc_ and *I*_LSP_ approach their maximum if (*ε*_M_ + 2*ε*_d_) approach zero, i.e., Re(*ε*_M_), and with positive and close-to-zero Im(*ε*_M_) could support strong LSPR intensities and giant local field enhancement for SERS enhancement in the ultraviolet, visible, and near-infrared spectral range, and for SEIRA enhancement in the mid-infrared range. Au, Ag, and Cu can work as plasmonic materials in the visible with low intrinsic losses. Besides the coinage metals, alkali metals (Li, Na, K, Rb, and Cs) can also work as plasmonic materials, but it is very challenging to prepare stable nanostructured surfaces composed of these chemically active materials^49^. Al is a typical plasmonic material in the ultraviolet region. Ga, In, Pt, Rh, their alloys, and some metallic nitrides such as TiN, MoN have also been explored as plasmonic materials in the visible. For SEIRA, typical plasmonic materials in the mid-infrared are III-V semiconductors, electron-doped graphene nanoribbon, nanodiscs, etc.^36,38,50^. With the LSP coupling in an Au or Ag nanoparticle dimer with a nanogap (the hotspots, as shown in the red dot in Fig. 4b), both local and radiation fields can be unprecedentedly boosted, which results in single-molecule SERS sensitivity.

In Fig. 4c, propagating SPP exists at the plasmonic metal/dielectric interface^51^. Typical structures supporting SPP are a dielectric prism/metal substrate structure, a grating-modified metal substrate, a continuous metal thin film, a metal nanowire, etc. A moderately enhanced local field can be generated at the metal/dielectric interface or at the edges or gaps of the grating arrays, although the incident light-SPP coupling efficiency could be as high as nearly 100%. Structures only supporting SPP cannot provide strong enough local field enhancement for SERS or SEIRA, however, coupled structures such as an Au thin film coupled with an Au nanoparticle can be built to support the SPP coupled with LSP for giant local field enhancement in the particle–substrate nanogap (Fig. 4d)^52,53^.

The LRE is widely present in a sharpened nanotip for strong local field enhancement at the apex of the tip^45^. The nanotip can be controlled to scan the sample surfaces with a scanning-probe microscope (such as atomic force microscope, scanning tunneling microscope, and shear force microscope) for nanoscale Raman or infrared spectroscopy. Usually, the samples are prepared as an ultrathin film on some metallic (e.g., Ag, Au, Cu, Pt, Pd) film, as a result, the Au or Ag nanotip and a metallic substrate form a coupled structure with a nanogap that supports the LSP for much higher local field enhancement in the nanogap (Fig. 4f). In TERS, by employing a silver tip coupled with a silver single-crystal substrate in low temperature and ultrahigh vacuum conditions, the spatial resolution of the TERS can be pushed down to nanoscale^54^. The huge EM enhancement in the gap between metal tip and substrate improves the sensitivity of TERS to a single molecule^55^.

In SEIRA, several types of metallic structures were developed for SEIRA-active substrates^35^. Among them, single-arm antenna (Fig. 4g) or dual-arm antenna with a nanogap (with gap size smaller than 20 nm, as shown in Fig. 4h) support strong local field enhancement at the ends of the nanorods^56^. Furthermore, metal strips with periodic grooves support spoof SPP for much higher local field enhancement in the grooves where the ultrahigh SEIRA sensitivity can be reached^50^.

### Coupling plasmonic substrates

Coupling plasmonic substrates supporting both LSP and SPP or LRE are named as coupling structures, which can further significantly increase the sensitivity of SERS and SEIRA. Coupled structures including nanoparticle–nanoparticle and nanoparticle–substrate (Fig. 4b, d, f, h, i), as well as the selection of the excitation wavelength and plasmonic materials jointly determine the ultrahigh sensitivity of SERS and SEIRA. For a long time, nanogap engineering is the basic and crucial task for ultrasensitive SERS or SEIRA-active structures^57^. In the following section, a series of coupled structures will be discussed, such as interparticle, particle–substrate coupled structures, and nanotip-substrate.

**Fig. 5:**
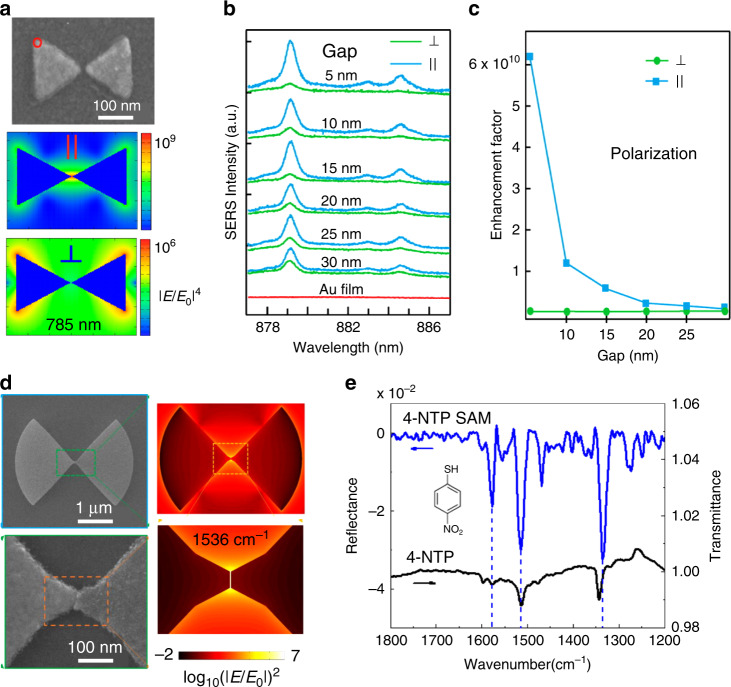
Dimers for SERS and SEIRA. **a** SEM image of prism dimers and simulated electric field contour plot of both polarizations. **b** SERS spectra of 2-naphthalenethiol SAM excited by 785 nm laser, and **c** SERS enhancement factor of prism dimers with varying gaps between prisms for both polarizations. Reproduced with permission from ref. ^60^. Copyright (2013) American Chemical Society **d** SEM and simulated electric field distribution of a bowtie antenna (at 1536 cm^–1^). **e** SEIRA of 4-NTP SAM on a single antenna. Reference spectra of solid-state 4-NTP are shown in black with prominent vibrations indicated by dashed lines. Reproduced with permission from ref. ^62^. Copyright (2017) American Chemical Society

The interparticle coupled nanostructures include nanosphere dimers or oligomers, a nanorod dimer, and a prism dimer^16,58,59^. As shown in Fig. 5a, a strong local EM field can be generated if the polarization of the incident light is parallel to the axis of the prism dimer^60^. The SERS enhancement factor under the parallel illumination strongly depends on the nanogap size and increases up to ten orders of magnitude once the nanogap size decreases to 5 nm (Fig. 5b-c). McMahon et al. demonstrated a universal behavior of |***E***_loc_/***E***_0_|^2^ in the gaps between closely spaced nanostructures, with gap size *a* of the form 1/*a*^*p*^ (*p* ≈ 1.2–1.5), which is weaker than the result expected based on simple antenna theory arguments of 1/*a*^2^. This feature was shown to occur “irrespective of the geometry of the nanostructures, and are applicable to both perfect conductors as well as metals that support LSP”^61^.

Along this direction, nano or combined nano-micro structures can also be fabricated as SEIRA-active substrates. As shown in Fig. 5d–e, Halas and coworkers fabricated a pair of microfans with a nanogap (3 nm in the gap size) on a gold reflector. The simulation shows that the |***E***_loc_/***E***_0_|^2^ in the gap reaches up to seven orders of magnitude, and the measured detection sensitivity on this antenna structure is up to 500 molecules (4-nitrothiophenol, 4-NTP)^62^. This strategy offers a new platform for analyzing the IR vibrations of minute quantities of analyte molecules and lends insight into the ultimate limit of single-molecule SEIRA detection. Notably, the dimension of a SEIRA metal substrate should be much larger than that of a SERS metal substrate although the shape of the two types of substrates could be very similar.

**Fig. 6:**
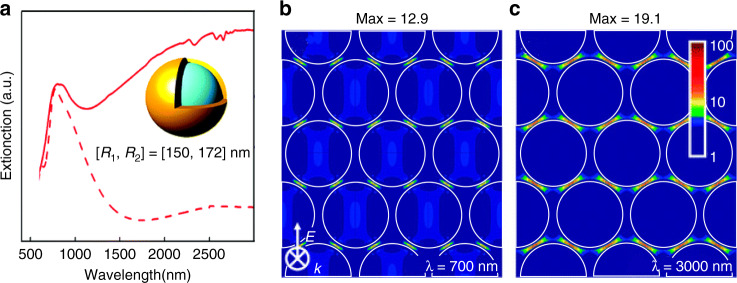
Multi-nanoparticles for SERS and SEIRA. **a** Extinction spectra of a typical hexagonal close-packed (hcp) Au nanoshell array (solid line) and that of an isolated nanoshell with the same size (dashed line). The inner radius of the core is 150 nm and the thickness of the gold shell is 22 nm. The separation between adjacent nanoshells is 8 nm. **b** and **c** The local electromagnetic field enhancements of an HCP Au nanoshell array at wavelengths of 700 and 3000 nm, respectively^65^. Reproduced **a**–**c** with permission from ref. ^65^. Copyright (2008) American Chemical Society

A self-assembled method has been employed to prepare SERS- and SEIRA-active substrates with high spot-to-spot reproducibility for maximizing the sensitivity. Liz-Marzán and coworkers assembled nearly perfect three-dimensional super crystals of Au nanorods as the SERS substrates with uniform electric field enhancement, leading to reproducibly high enhancement factor^63^. Halas and coworkers developed an Au nanoshell with a dielectric core and a gold shell, and assembled the nanoshells into an array. As shown in Fig. 6a, a single nanoshell shows a resonance band in the visible, while the nanoshell array with interparticle nanogaps^64,65^, shows a resonant band in the near-infrared and an additional broadband resonance in the mid-infrared. The authors assigned the near-infrared band to the hybridization of the multipolar plasmon resonances of individual nanoshells, which can be used for enhancing SERS signals (Fig. 6b), and assigned the broadband resonance in the mid-infrared to the dipolar resonances of multiple nanoshells, which can increase the sensitivity of SEIRA (Fig. 6c). Interparticle coupled structures show strong SERS or SEIRA sensitivity, but it is difficult to precisely control the size of the interparticle nanogap^58^. Plasmonic intra-nanogap particles with an interior gap are designed to improve the reproducibility of nanogap^66^. However, the inter- and intra-nanogap particle structures are not well suited for surface analysis of many materials. For example, widely used materials such as silicon wafers or ceramics cannot be squeezed into the extremely tiny and narrow regions of the hotspots formed by the interparticle or intraparticle nanogaps. Thus, it is necessary to novel measurement modes to perform surface analysis of general materials^17^.

**Fig. 7:**
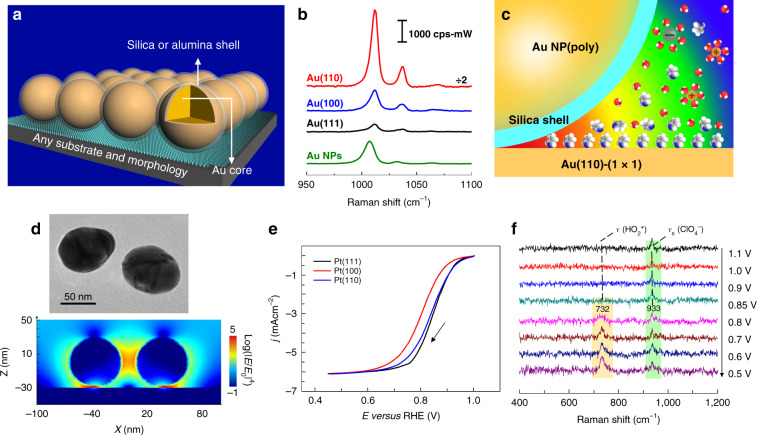
SHINERS for surface detection. **a** SHINERS on a substrate^31^. **b** SHINERS spectra of pyridine adsorbed on Au(111), Au(100), and Au(110) at 0.00 V. Solution: 10 mM pyridine + 0.1 M NaClO_4_. SERS spectra of pyridine on bare 55 nm gold NPs at different potentials. **c** Schematic diagrams of the SHINERS experiments. The EM field strength is represented by the following color code: red (strong) and blue (weak).^67^ **d** TEM and simulated images. 3D-FDTD simulations of four SHINs with a model of a 2 × 2 array on a Pt substrate. *E* and *E*_0_ represent the localized electric field and the incident electric field, respectively. **e** Polarization curves of the ORR process at three Pt(hkl) RDEs in oxygen-saturated 0.1 M HClO_4_ solutions; the rotation rate was 1600 r.p.m. and the scan rate was 50 mV s^−1^. j and *E* represent the current density and potential, respectively. **f** EC-SHINERS spectra of the ORR system at a Pt(111) electrode surface in a 0.1 M HClO_4_ solution saturated with O_2_^74^

In 2010, our group invented SHINERS^31^. In SHINERS (Fig. 7), the shell-isolated NPs (SHINs) are composed of plasmonic Au or Ag cores with ultrathin (1–5 nm) chemically and electrically inert shells (for example, of SiO_2_, or Al_2_O_3_). The major three advantages of SHINERS are as follows: (1) The ultrathin yet pinhole-free shells separate the Au or Ag cores from the material surface (and environment) thus ensuring that there is almost no chemical interference from the cores to the probed substrate surface; (2) the chemically inert shell effectively avoids interparticle and particle–metal substrate fusion that may be caused by the strong laser beam, which significantly improves the stability of the NPs and the probe structures; (3) the shell thickness can be used to control the Au or Ag core particle–substrate nanogap size, and consequently determines the particle–substrate EM coupling^21^. In particular, several shell-isolated nanoparticles form a cluster on the metal substrates, which can effectively couple with the incident light to generate a strong local field on the atomically flat metal surfaces^67,68^. With the interesting EM coupling in SHINs-substrate coupled systems, we have revealed many fundamental electrocatalytic mechanisms on various single-crystal electrodes with different facets^21,67,69–75^, such as oxygen reduction reaction on Pt(*hkl*)^76^, and water structure on Au(*hkl*)^74^.

In addition to the interparticle nanogaps, and particle–metal substrate nanogaps, tip-metal substrate nanogaps are usually designed for ultrahigh sensitivity in TERS. Dong and coworkers employed a Ag tip as a scanning tunneling probe to approach a Ag(111) single crystal under low temperature and ultrahigh vacuum conditions. In this way, TERS with single-molecule resolution and even subnanometer spatial resolution can be observed^77^. Tip-enhanced infrared nanospectroscopy via molecular expansion force detection can also be obtained using an Au tip that approaches a monolayer molecule on an Au film^78^. Either in TERS or tip-enhanced infrared nanospectroscopy, tip-metal substrate not only supports LSP but the strong LRE in the nanogap. LSP provides a broad local field (about 10 nm) and LRE further improves the confinement of the local field to a subnanometer level making single-molecule imaging possible. The picocavity demonstrated by Baumberg’s group in 2016 illustrates the confinement of LRE^79^. Monolayer molecule is self-assembled into the gap between Au nanoparticle and Au mirror (NPoM). Thus a 1 nm nanocavity is formed in NPoM. At ULT and UHV, stokes and anti-stokes Raman spectroscopy from NPoM blinking when the power of excitation laser exceeding a threshold. This phenomenon is successfully explained by the picocavity, which is formed only when the Au atom motivated by the laser’s heat jumps into the nanocavity in NPoM. The result of the numerical simulation reveals that the atomic protuberance in nanocavity will induce the LRE in atomic scale and stronger confinement of EM field, namely the picocavity, is formed in nanocavity. When the position of picocavity relative to the internal position of a single molecule changes with the activated Au atom, Raman spectra blinking at the different vibrational modes of the molecules. In 2019, Apkarian’s group formed picocavity in the nanocavity between a Ag tip and Cu single crystal with a similar principle. With the atomic LRE, different vibrational modes of single molecule were imaged successfully^80^. It is worth noting that the atomic LRE, UHV, and ULT are all important to realize the imaging of submolecule^81–85^. Although atomic LRE also exists in traditional TERS, the temperature and pressure make the atomic LRE unstable, which makes TERS spectra always blinking.

By employing nano/micro-optical designs, SERS enhancement factor has been improved to 8–10 orders of magnitude in the inter-/intra-particle nanogap substrates, and SEIRA enhancement factor has been improved by seven orders of magnitude in the inter-triangles/fans nanogaps. Comparing with structures only supporting SPP, LSP, or LRE, coupled plasmonic structures are more efficient to support stronger local field in the SERS and SEIRA substrates with a large enhancement factor. One reason is that in the coupled structure the maximum local field is roughly determined by the product of two or more effects. For example, in the nanodimer of a triangle substrate LSPs and the LRE of the two triangles are excited independently. Thus, there is a chance for us to excite both LSP and LRE simultaneously, and the final EM enhancement in the nanogap on such substrates is the product of two triangle’s LSPs and LRE. It is worth noting that there exists always the interaction between two triangle’s LSPs which can further improve the local field enhancement in the nanogap. The fundamental limiting factor for the EM enhancement is the quantum tunneling effect in the nanogap^86,87^. Along with the EM enhancement, the possibility of the electron’s ‘jump’ is also improved from one side to the other side of the nanogaps. The quantum tunneling effect will decrease the EM enhancement in the nanogap by electron’s ‘jump’. Thus, the nano/micro-optical designs for ultrasensitive SERS and SEIRA should consider the balance between the EM enhancement effects and quantum tunneling effect, which is still a challenge. In addition, it is necessary to note that single-molecule SERS is only applicable to limited molecular systems that have large Raman cross-section, and single-oscillator SEIRA has not yet been achieved. How to further improve the sensitivity of SERS and SEIRA for general molecules on general material surfaces beyond few noble metals such as Au, Ag, Cu is still challenging^88^.

## Macro-optical designs based on the nano/microstructured substrates

The macro-optical designs including excitation and collection optics are of great importance for ultrasensitive SERS and SEIRA measurements. The two main reasons are as follows: (1) The excitation optics including incident angles, polarization states, and beam shapes determines the overall coupling efficiency between the incident light and SERS or SEIRA-active nano/microstructures having the strongest localized EM field at hotspots. (2) The Raman scattering photons in SERS are usually directionally emitted, thus the collection optics determine how much Raman scattering generated by the molecules in the hotspot could be transmitted to the detector with the molecular emitter-nanostructure coupling. However, the general optics used in micro-Raman and IR spectrometer only provide linearly polarized laser at a normal incident angle and collect the light transmitting inside the solid angle determined by the objective’s numerical aperture (NA).

From Eqs. 1 and 2 both SERS and SEIRA are not only determined by substrates but the collection efficiency Ω of the macro-optics. The collection efficiency Ω can be further expanded to a general expression,

18 $${\Omega} \propto \left( {\frac{{S_{{{{\mathrm{exci}}}}}M_{{{{\mathrm{e}}}} - {{{\mathrm{e}}}}}}}{{{\Omega}_{{{\mathrm{e}}}}}}} \right) \cdot \left( {{\Omega}_{{{\mathrm{c}}}}S_{{{{\mathrm{scat}}}}}M_{{{{\mathrm{c}}}} - {{{\mathrm{s}}}}}} \right)$$

where ${\Omega}_{{{\mathrm{e}}}}$ is the solid angle of the excitation laser. ${\Omega}_{{{\mathrm{e}}}}$ is determined by the excitation optics and excitation laser’s divergence angle. $S_{{{{\mathrm{exci}}}}}$ is to evaluate the property of substrate’s directional excitation. $M_{{{{\mathrm{e}}}} - {{{\mathrm{e}}}}}$ is to evaluate the match between excitation laser and substrate. ${\Omega}_{{{\mathrm{c}}}}$ is the solid angle of the collection optics. $S_{{{{\mathrm{scat}}}}}$ is to evaluate the property of substrate’s directional emission. $M_{{{{\mathrm{c}}}} - {{{\mathrm{s}}}}}$ is to evaluate the match between collection optics and the substrate. From Eq. 18, an ideal excitation and collection of the optical system for SERS and SEIRA need a good concentration of the excitation light energy in the solid angle, a strong directional excitation and radiation properties of the substrate, higher collection efficiency, and a good match between the substrate and the excitation/collection optics.

**Fig. 8:**
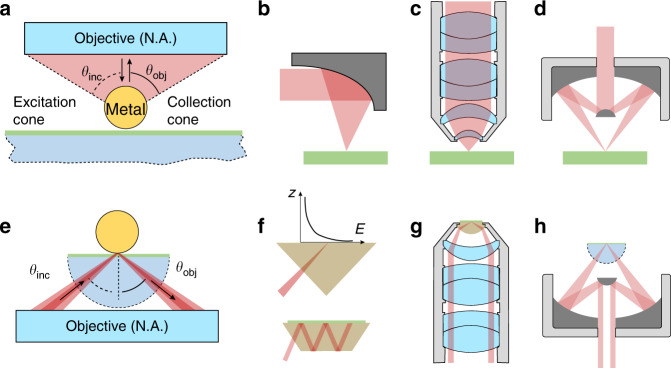
Excitation and collection optics for SERS and SEIRA. **a** Traditional excitation and collection cone for SERS and SEIRA. **b** Reflective focus mirror, **c** traditional refractive, and **d** reflective objectives to excite and collect SERS and SEIRA. **e** Fine designed excitation and collection hollow cone for SERS and SEIRA. **f** Prism and waveguide-based excitation optics, **g** prism integrated refractive, and **h** reflective objectives to excite and collect SERS and SEIRA signals

In normal Raman and IR microscopes as shown in Fig. 8a–d, excitation and collection optics are based on reflective focus mirror, refractive or reflective objectives. The NAs of them are designed as high as possible to collect most enough of the signals from the sample surface. The angle of incidence (AOI) covers ranges from 0 to ${{{\mathrm{arcsin}}}}\left( {{{{\mathrm{NA}}}}/n} \right)$ in the excitation cone, where *n* is the refractive index of the environmental medium. In a typical SERS system with a nanoparticle on a flat substrate, incident beams at a higher angle are more efficient to generate a strong local EM field in the nanogap between nanoparticle and substrate. Moskovits et al. experimentally and theoretically demonstrated that SERS from Au NP/3 nm-Silica/Au mirror substrate has the strongest intensity at an angle of incident of 60 degrees^89^. Our group further simulated nanoparticles on substrates of different materials and got similar results^88^. However, in normal Raman and IR microscopies, the excitation laser is mainly distributed around the optical axis, only a fraction of the excitation laser’s energy is distributed at high incident angles (Fig. 8a) to excite the LSP in the nanogap between nanoparticle and substrate. Notably, the excitation and collection beams should be designed as a hollow cone^90–93^, in which the incident beam is distributed within a narrow range of solid angles to improve the excitation efficiency of SERS and SEIRA (Fig. 8e). Figure 8f–h shows several configurations with ATR prisms coupled with refractive or reflective objectives to support the excitation hollow cone^53,94,95^.

### ATR optics to directional excite SERS and SEIRA substrates

**Fig. 9:**
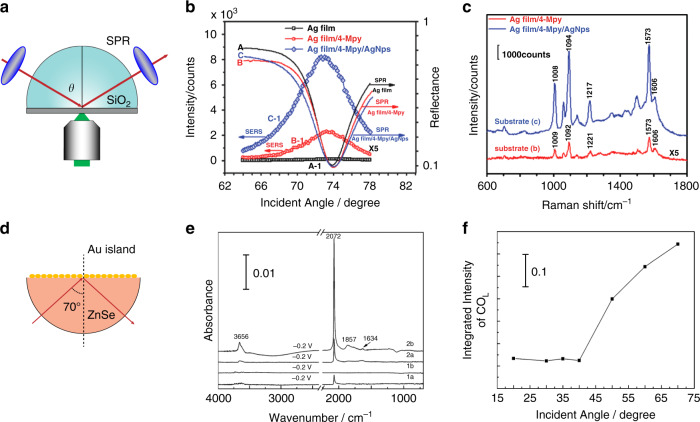
ATR-SERS and ATR-SEIRA optics for LSP and SPP coupling. **a** Schematic diagram of a Kretschmann SPR sensor. **b** AOI-dependent SERS spectra on silver film and nanoparticle-modified silver film modified by 4-Mpy single-layer molecule. **c** The SERS spectra of 4-Mpy on silver film and nanoparticle-modified silver film at an incident angle of SPR angle^52^. Adapted **a**–**c** with permission from ref. ^52^ Copyright (2011) Royal Society of Chemistry. **d** Schematic diagram of ATR-SEIRAS configuration containing a ZnSe hemicylindrical prism and metal island film. **e** ATR-SEIRA spectra of a Pt film electrode in CO-saturated 0.1 M HClO_4_ at −0.2 V. Spectra 1a and 2a were recorded at the incidence angle *θ* = 20°, and spectra 1b and 2b at *θ* = 70° with ZnSe/air/Si (1a and 1b) and ZnSe/water/Si (2a and 2b) windows, respectively. **f** SEIRA band intensity for CO_L_ on the Pt electrode as a function of *θ*, measured with the ZnSe hemicylindrical prism^94^. Reprinted **d**–**f** with permission from ref. ^101^ Copyright (2008) American Chemical Society

The nanoparticle-on-metal film structure can be excited by a traditional optical configuration as shown in Fig. 8a^67^. The structure can also be excited through the ATR prism/metal film/nanoparticle configuration as shown in Figs. 8e and 9a^53^. At the critical angle at which the SPP at the film/air interface is on resonance, the SERS intensity at the film surface with or without the coupled nanoparticles reaches the maximum (Fig. 9b, c). Researchers named this configuration as SPR-SERS configuration. Around 2010, several groups reported a SPR-SERS spectrometer to investigate the SPP-based SERS^96–99^. Xu and coworkers have investigated the incident angle-dependent SERS in the SPR-SERS configuration^52,100^. Excitation optics use a long focal length, low NA lens (NA = 0.15) to focus linearly polarized excitation light through a half-cylinder prism on the lower surface. A precision goniometer (accuracy <0.005°) controls the rotation of the excitation optics around the axis of the half-cylinder. As shown in Fig. 9a, by simultaneously measuring the SERS and SPR spectra of analytes, the authors found that the strongest SERS signals were measured at the vicinity of the resonance angle. The enhancement factor was about 2.0 × 10^6^. The effective coupling of the SPP and LSP provides hundreds of “hot spots” between nanoparticles and the metal film, which has been confirmed to be responsible for the additional enhancement of the SERS signals. Notably, evanescent field-based directional excitation is also an effective way to improve the enhancement of SEIRA (Fig. 9d–f)^101^. Such SEIRA measurements can be performed using a metal array substrate deposited onto the surface of a zinc selenide (ZnSe) prism.

### SERS directional emission from NP aggregates

**Fig. 10:**
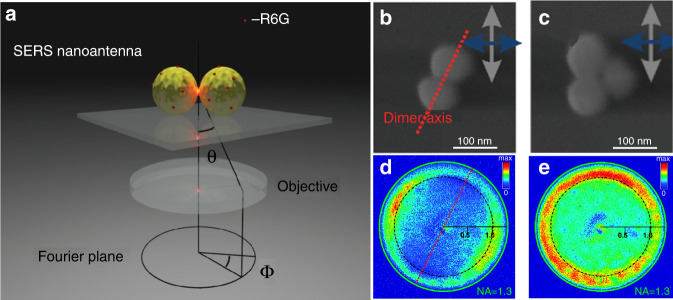
Angle-resolved SERS optics for NP aggregations. **a** Scheme of the angle-resolved SERS detection. **b** SEM image of the dimer antenna. **c** SEM image of the trimer antenna. **d** SERS Fourier image of gold dimer nanoantenna. **e** SERS Fourier image of gold trimer nanoantenna^91^. Reprinted with permission from ref. ^91^. Copyright (2011) American Chemical Society

Shegai et al. investigated the angle-dependent distribution of SERS signals radiated from the dimer and trimer antennas using Fourier plane SERS imaging^90,91^. As shown in Fig. 10a, an aggregate was positioned within the field of view of the objective and excited by the laser. The scattered SERS photons were then collected by the same objective. The angular distribution of SERS radiation could be directly monitored in the Fourier plane of the optical microscope. The cylindrical coordinates were used to describe the Fourier image so that the radial coordinate scales in proportion to ∼sin *θ*, and the tangential coordinate scales as *φ*, where *θ* and *φ* are defined in Fig. 10a. With a NA = 0.7–1.3 oil immersion objective, SERS from dimer and trimer decorated with Rhodamine-6G dye molecules was imaged in Fig. 10b, c. In Fig. 10d, the image is symmetric with respect to the dimer axis, depicted as a red dotted line. The green circle in the Fourier image indicates NA_max_ = 1.3 of the objectives, which determines the maximum collection angle. The radial coordinate in the Fourier image is proportional to NA, so the position of the green circle unequivocally determines the angle *θ* in the Fourier plane. To make the reading of the Fourier data easier, the position of NA = 1.0 and 0.5 is also shown. The most intense areas are observed at angles exceeding the critical angle (*θ*_c_ = 41.5° or NA = 1) for an air–glass interface. The corresponding SERS Fourier image is shown in Fig. 10d. The fact that the axial symmetry is lost in a trimer in comparison to a dimer nanoantenna is reflected in the Fourier image. But the maximum radiation is observed at angles exceeding the critical angle as the same results in Fig. 10e. The results in Fig. 10 show that most of the SERS signals were emitted at angles exceeding the critical angle. Thus, the collection optics are important when measuring and designing SERS substrates.

### PERS objective for directional excitation and collection in one setup

**Fig. 11:**
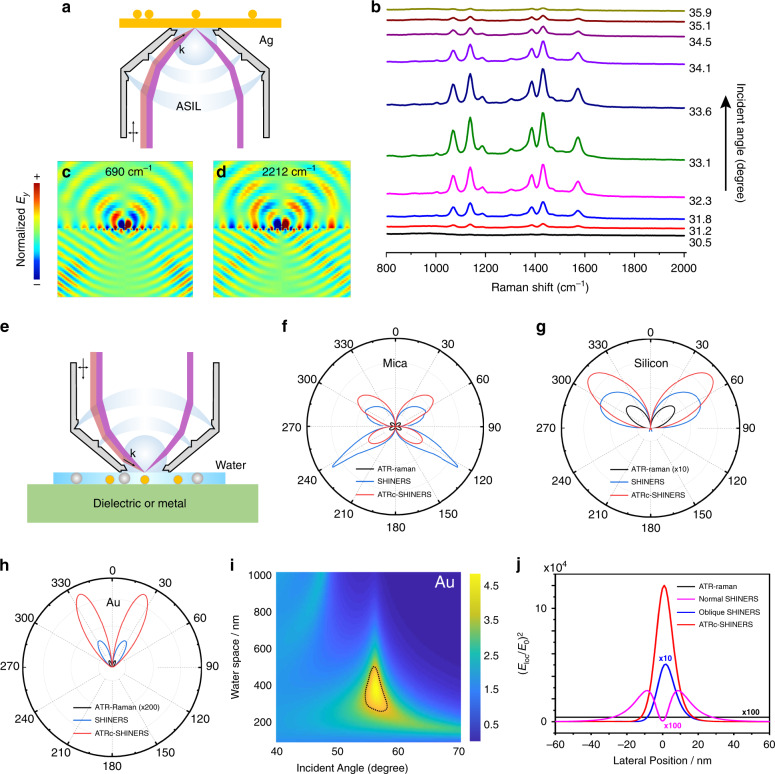
Excitation and collection optics for nanoparticle on a film. **a** Custom-designed objective for excitation and collection of nanoparticle on a film for SERS. *p*-ATP monomolecular layer was assembled on the surface of silver film. **b** AOI dependent SERS spectra of NOF decorated by *p*-ATP monomolecular layer. **c** and **d** Directional emission of SERS signal (690 cm^−1^ and 2212 cm^−1^) from nanoparticle on a film substrate. **e** Schematics of the ATRc-SHINERS optics^95^. Directional emission of SERS signal in Otto ATR-Raman, SHINERS and ATRc-SHINERS configurations from **f** Mica and **g** Si substrates. **h** Directional emission of SERS signal in ATR-Raman, SHINERS and ATRc-SHINERS configurations from an Au substrate. The Raman-scattered wavelength is 700 nm, corresponding to the Raman shift at 1,500 cm^−1^. **i** Incident angle and thickness of water space-dependent electrical field on the Au surface. Excitation wavelength is 633 nm. **j** Local field enhancement with different excitation optics^53^

In 2017, Xu’s group invented an integrated plasmon-enhanced Raman scattering (iPERS) spectroscopy^95^. This setup integrates the SERS substrates and Raman spectrometers in one setup via a custom-designed PERS objective^102^. The PERS objective is an aplanatic solid immersion lens (ASIL) with a large NA (1.65) designed according to the theory of aplanatic lenses^103^. Compared to the hemispherical prisms commonly used in ATR optics, a high refractive index (*n* = 1.92) super-hemispherical prism was adopted in the ASIL as the very front lens. The SERS substrate is located at the top of the super-hemispherical prism. ASIL not only collects more Raman scattering from the SERS substrate but also focuses the excitation beam from the lens edge to the substrate to satisfy the large incident angle demanded by the SERS substrate. The ASIL well unites the local field enhancement and far-field emission with localized and propagating SPP coupling. As shown in Fig. 11a–d, the excitation optics are linearly and controllably shifted toward the center of the optical axis in the direction perpendicular to the ASIL objective optical axis. In the ATR prism section, AOI of excitation light is adjusted to optimize the excitation angle with the linear movement of the excitation optics. Simultaneously the large NA of the iPERS can collect almost all the SERS signals radiating from the enhanced substrate. With optimization of the incident angle, the local field can be amplified by ten orders of magnitude on account of the simultaneous excitation of quadrupolar and dipolar resonance modes. The iPERS allows for higher excitation efficiency and a full collection of the directional radiation of the Raman-scattered signal in an inverted way, which exhibits a practical possibility to monitor plasmonic photocatalytic reactions in nanoscale and a bright future on interfacial reaction studies.

SHINERS is an important PERS substrate because of its reproducibility and reliability but short of sensitivity. To improve the sensitivity, as shown in Fig. 11e our group designed an ATR-cascading SHINERS (ATRc-SHINERS) optics based on an ATR-cascading nanostructure-enhanced Raman spectroscopy on flat surfaces^53^. The design of ATRc-SHINERS optics is based on the concept of cascading EM field from macro to nano scale. ATRc means the macro-optics that are lens supporting ATR mode as the first step to focus EM field from macro to micro scale. Comparing with the direct excitation in the normal Raman microscope, the ATR optics in ATRc-SHINERS guide and focus the incident light at the bottom lens surface as shown in Fig. 11e. Once the ATR condition is fulfilled when the incident angle is greater than the critical angle, the evanescent field on the surface of the ATR prism increases up to 1–2 orders of magnitude. When SHINs are located into the evanescent field, the local field around SHINs is boosted up to five orders of magnitude as shown in Fig. 11j. The EM intensity of SHINERS, by contrast, is only three orders of magnitude when directly excited in free space. Thus ATRc-SHINERS can effectively harvest the incident light, thereby boosting the local optical field of the incident light than normal optics in a Raman microscope. In addition, the emission of SHINERS on different flat surfaces is highly directional. As shown in Fig. 11f–h, no matter on dielectric surfaces like mica, silicon, or gold surface, ATRc-SHINERS optics has the strongest SERS signal than ATR-Raman and normal SHINERS. This directional emission can be collected with the noise scattering in other solid angle blocked, and the sensitivity of SHINERS in ATRc- SHINERS optics will be improved further. With moderately increasing the radiation field of the Raman-scattered signals, one can gain 1–2 additional orders of magnitude in Raman enhancement larger than that of present nanostructure-enhanced Raman spectroscopy on flat surfaces both on metallic and nonmetallic flat surfaces, which are otherwise SERS-inactive.

In 2020, Baumberg’s group developed cascaded nano optics structures incorporating both refractive and plasmonic optics, by creating SiO_2_ microlens fused to Au nanoparticle^104^. They routinely achieved significant improvements in SERS efficiencies, with (single-wavelength) emissions reaching 10^7^ counts mW^−1^ s^−1^ and 5 × 10^5^ counts mW^−1^ s^−1^ molecule^−1^, for enhancement factors >10^11^. The high optical efficiency and field enhancement allow for spectra to be collected at submicrowatt laser powers and provide unrivaled signal-to-noise ratios, reaching >10^3^ to 1 for only 250-μJ laser dose. In their structures, the combined effects of nanolensing, reexcitation, and symmetry breaking as well as a light concentration through nanoscale reorientation of the AuNP enhanced the incoupling and outcoupling of laser-driven SERS signals from molecules assembled inside the integrated nanogaps.

### Electrochemical and fiber optics for tip-based systems

**Fig. 12:**
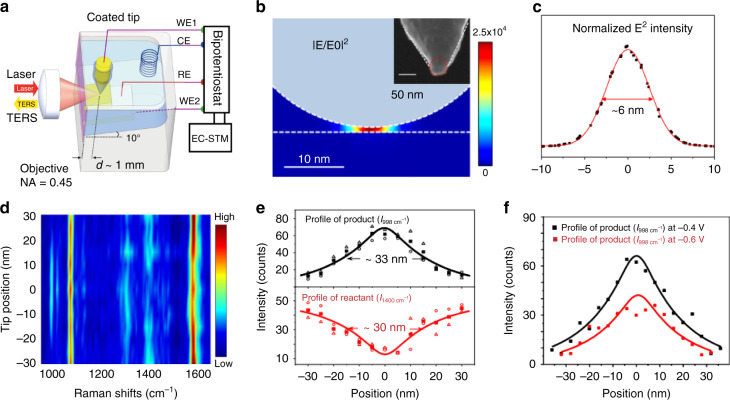
Macro-optics for EC-TERS. **a** Schematic illustration of the EC-TERS^105^. **b** Calculated plasmonic electric field (*E*^2^) distribution when the Ag tip is positioned on the Au surface. Inset: scanning electron microscope image of the Ag tip. **c** Calculated *E*^2^ profile on the substrate surface in the plasmonic gap. Red line is fitted using a Gaussian function. **d** Color-coded intensity map of the line-trace TERS spectra across a reaction region. The reaction was induced at the sample potential of −0.4 V under a laser power of 0.7 mW. The line-trace TERS spectra were acquired with the sample potential at −0.7 V. Bias: 100 mV, I_tunneling_: 800 pA. **e** Plots of intensities of the 998 cm^−1^ peak (top) and 1400 cm^−1^ peak (bottom) with the tip position. The open triangle, circle symbols represent the intensities in the trace and retrace spectra. The solid square symbols represent the average intensities of the trace and retrace spectra. **f** Profile of the reaction induced at −0.6 and −0.4 V for comparison^106^

TERS provides fingerprint molecular information at nanometer resolution. For TERS in ambient or UHV conditions, the objectives can be directly coupled with the tip. However, it is challenging to couple the objective with the tip in solution for TERS working in liquid due to the distorted beams from air to liquid. For electrochemical TERS (EC-TERS), a side-illumination configuration should be employed as the working electrode is opaque. Consequently, it is impractical to use a high NA objective (usually oil- or water-immersion) with a short working distance. To overcome the limitation in EC-TERS, Ren and coworkers designed an EC-STM optical cell by tilting the single crystal to about 10°, so that the laser can be properly focused onto the tip and the Raman signal can be collected with high efficiency^105,106^, as shown in Fig. 12a. 10° is a sweet balance to keep the high imaging quality of STM and high collection efficiency of the Raman system, without much modification of the STM system. Benefitted from this design, the optical path will not change compared with that of tilted illumination even if there is the evaporation of the electrolyte during the EC-TERS measurement, allowing a long-time measurement. They used this EC-TERS setup to in situ monitor a SP-driven decarboxylation and resolved the spatial distribution of hot carriers with a nanometer spatial resolution. Theoretical simulation of the Ag tip using the geometry parameters in the experiment was carried out to calculate the distribution of local fields in the plasmonic gap between the tip and substrate (Fig. 12b, c). As the intensity of the electric field decreases dramatically inside the metal, the local field distribution on the top surface of the substrate was used as the maximum of the one inside the metal. The FWHM of the local field distribution is about 6 nm (Fig. 12c), which is much smaller than the size of the reaction region. As shown in Fig. 12d, e, when the tip is scanned from the perimeter of the reaction region toward its center, the peak intensities of the product at 998 and 1020 cm^−1^ gradually increase and reach a maximum at the center, while the peak intensity of the reactant at 1400 cm^−1^ shows the opposite trend. In EC-TERS, the energy of hot carriers can be conveniently tuned by changing the potential of the substrate. The transport distance is the distance from the center of the reaction region to the position where the reaction stops in real space. The transport distance is determined by the energy of the hot carriers and can be revealed by the region size of the reaction product. When the potential was shifted from −0.4 to −0.6 V, the energy barrier for OH− oxidation was increased from about 1.66 to 1.86 eV (relative to the Fermi level) and reduced the reactivity at the reaction region (Fig. 12f). The transport distance estimated using the data in Fig. 12f is ~17 nm at −0.6 V and ~20 nm at −0.4 V for the hot holes with energies higher than 1.86 and 1.66 eV, respectively, relative to the Fermi level. Thus the nanoscale reaction region beneath the tip with EC-TERS imaging of a plasmon-driven decarboxylation reaction by turning on and off the reaction with potential control was visualized. The transport distance for the reactive hot holes in real space was obtained.

**Fig. 13:**
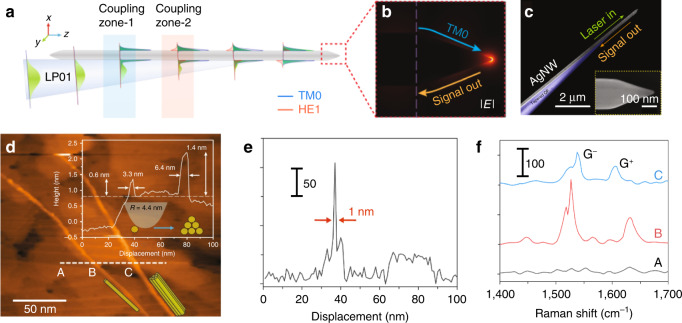
Fiber-coupled TERS tip. **a** The phase-matching zones for TM_0_ and HE_1_ modes are separated for the selective excitation of the former. **b** Simulation of the nanofocusing at the AgNW tip (tip angle 37°), with TM_0_ input from the left. The light wavelength is 532 nm for all calculations. **c** False-color SEM images of a fabricated AgNW-OF probe. The OF tip region (blue) is uncovered for the selective and effective excitation of the TM_0_ SPP on a sharp-tip silver nanowire (200 nm in diameter, ~5 nm in tip radius). Inset of **c**: enlarged view of the AgNW tip. **d** STM topographic image of SWCNTs on an Au film. Top inset: cross-sectional profile along the dashed line. Bottom inset: the possible configurations of the bundle. **e** Intensity of the Raman peak at 1526 cm^−1^ along the white dashed line in (**d**). A, B, C in **d** and **e** indicate the position of the Raman spectra in (**f**). Reprinted with permission from ref. ^107^ from Springer Nature: Nature Photonics, Copyright (2019)

The optical design of directional excitation and collection is also applicable in TERS. As shown in Fig. 13a–c, Liu and Yan, and coworkers developed an all-fiber-coupled geometry by coupling incident and scattered light with a fiber-Ag nanowire (AgNW) hybrid probe^107^. By optimizing the taper angle of the optical fiber to 7°, wavevectors of LP_01_ in the optical fiber and SPP mode TM_0_ in the AgNW match in coupling zone 1. SPP mode TM_0_ in the AgNW is a radially polarized mode propagating along AgNW and adiabatically focusing at the tapered tip. SPP mode HE_1_ in the AgNW also can match with LP_01_ in coupling zone 2, but too much power will be dissipated when SPP mode HE_1_ propagating along AgNW. The nanofocusing effect in the hybrid fiber-AgNW provides high efficiencies in both incident excitation and signal collection, which is capable of both light delivery and spectrum collection with nanoscale spatial resolution. The two-step sequential nanofocusing achieves an external nanofocusing efficiency of ~50% in the visible range. Integrating the hybrid fiber-AgNW with a basic portable scanning tunneling microscope, the lens-free TERS was realized. Figure 13d shows an STM topographic mapping of single-walled carbon nanotubes (SWCNT). The left bundle has a height of 0.6 nm and a full-width at half-maximum (FWHM) of 3.3 nm, while the right one has a height of 1.4 nm and an FWHM of 6.4 nm. The TERS spectra on the SWCNT is shown in Fig. 13f, exhibiting two clear bands at ~1540 cm^−1^ and ~1600 cm^–1^, corresponding to the G^+^ peak and G^−^ peak, respectively. Figure 13e plots the intensity of the G^−^ peak as a function of the position. An FWHM of 1 nm was achieved on the single SWCNT and the sensitivity is down to 208 c.p.s. (counts per second). The fiber-based nanofocusing technique with high performance is a case to support the importance of the coupling from macro to micro-optical design.

## Conclusion and outlook

The advent of SERS and SEIRA inherits the advantages of the high spectral resolution of Raman and IR spectroscopy but still suffers from the disadvantage of low detection sensitivity with small molecules. By rationally designing coupled structures with interparticle nanogap and particle–metal film nanogap with ultranarrow gap size down to 1 nm, the SERS sensitivity has been improved up to single-molecule sensitivity. However, SERS and SEIRA strongly depend on the nanogap and substrate materials. It limits their applications for broad fields in which plasmonic nanogap is absent or the nanogap cannot be pushed down to 1–3 nm. The single or coupled nano/microstructures have their preferable incidents polarization and beam shapes, also have their preferable scattered polarization and emission patterns. Thus, macro-optics including excitation and collection optics can be designed to fit the preference of the nano/microstructures with high coupling efficiency. We have introduced several macro-optical designs such as ATR prism, hollow-cone beams, fiber-coupled Ag nanowire tip for ultrasensitive TERS. We believe that the macro-optic design is just in its early stage. Rational macro-optical designs and fabrications for the specific nano/microstructures for SERS and SEIRA are highly demanding in the future.

In addition to the coupling between nano/micro-optics and macro-optics, the finer coupling between nano/micro-optics and subnanometer-optics should also be explored. Very recently, Apkarian’s and Dong’s groups reported that the integrated subnanometer tip in the nanogap forms a picocavity, which contributed ultrahigh angstrom-resolved spatial resolution in TERS^79,80,108^. For macro-optics, scanning microsphere microscopy, which has gained widespread attention in recent years, converges excitation light into a nanojet through a micro-scale dielectric sphere^109^. The combination of scanning microspheres with plasmon-enhanced substrates is expected to widen the application area of SERS^104^.

Regarding the interaction of light with matter, a key feature of SEIRA is that the length scale spans more than three orders of magnitude from several wavelengths (tens of micrometers) in incident focusing spots to several nanometers in the nanogap. Typically, the coupling efficiency is low and as a consequence, ultrahigh sensitivity down to single-oscillator detection has not yet been achieved. Therefore, the integrated macro-micro-nano-optic design is promising for SEIRA. Besides, optical designs fitting some light sources with high beam quality, especially infrared sources such as free-electron lasers, are also important for ultrasensitive SEIRA and nanoIR in the future.

Macro-optical design should not only fit for the nano/micro-optical design, but also for the practical applications. In contrast to TERS, nanoIR spectroscopy is difficult to work in an aqueous environment. Very recently, nanoscale infrared spectroscopy and imaging in liquid environments have been partially overcome by the ATR prism-coupled optics^110,111^. However, the sample should be prepared as an ultrathin sheet. Further efforts on the macro-optic design will advance infrared spectroscopy for direct molecular-level studies of a wide range of application systems in electrochemistry, material science, life science, etc.

## References

[1] Sheppard, N. The historical development of experimental techniques in vibrational spectroscopy. in *Handbook of Vibrational Spectroscopy* (eds Chalmers, J. M. & Griffiths, P.R., 2006).

[2] Chabal YJ (1988). Surface infrared spectroscopy. Surf. Sci. Rep..

[3] Fleischmann M, Hendra PJ, McQuillan AJ (1974). Raman spectra of pyridine adsorbed at a silver electrode. Chem. Phys. Lett..

[4] Albrecht MG, Creighton JA (1977). Anomalously intense Raman spectra of pyridine at a silver electrode. J. Am. Chem. Soc..

[5] Jeanmaire DL, Van Duyne RP (1977). Surface Raman spectroelectrochemistry: part I. Heterocyclic, aromatic, and aliphatic amines adsorbed on the anodized silver electrode. J. Electroanal. Chem. Interfacial Electrochem..

[6] Moskovits M (1978). Surface roughness and the enhanced intensity of Raman scattering by molecules adsorbed on metals. J. Chem. Phys..

[7] Creighton JA, Blatchford CG, Albrecht MG (1979). Plasma resonance enhancement of Raman scattering by pyridine adsorbed on silver or gold sol particles of size comparable to the excitation wavelength. J. Chem. Soc., Faraday Trans. 2: Mol. Chem. Phys..

[8] Hartstein A, Kirtley JR, Tsang JC (1980). Enhancement of the infrared absorption from molecular monolayers with thin metal overlayers. Phys. Rev. Lett..

[9] Aroca, R. Surface-Enhanced Vibrational Spectroscopy (John Wiley & Sons, Ltd, Hoboken, 2006).

[10] Osawa M, Ikeda M (1991). Surface-enhanced infrared absorption of P-Nitrobenzoic acid deposited on silver island films: contributions of electromagnetic and chemical mechanisms. J. Phys. Chem..

[11] Osawa M (1997). Dynamic processes in electrochemical reactions studied by surface-enhanced infrared absorption spectroscopy (SEIRAS). Bull. Chem. Soc. Jpn..

[12] Bjerke AE, Griffiths PR, Theiss W (1999). Surface-enhanced infrared absorption of CO on platinized platinum. Anal. Chem..

[13] Nishikawa Y (1993). Silver island films for surface-enhanced infrared-absorption spectroscopy–effect of island morphology on the absorption enhancement. Vib. Spectrosc..

[14] Kneipp K (1997). Single molecule detection using surface-enhanced Raman scattering (SERS). Phys. Rev. Lett..

[15] Nie SM, Emory SR (1997). Probing Single molecules and single nanoparticles by surface-enhanced Raman scattering. Science.

[16] Xu H (1999). Spectroscopy of single hemoglobin molecules by surface enhanced Raman scattering. Phys. Rev. Lett..

[17] Ding SY (2016). Nanostructure-based plasmon-enhanced Raman spectroscopy for surface analysis of materials. Nat. Rev. Mater..

[18] Ding SY (2017). Electromagnetic theories of surface-enhanced Raman spectroscopy. Chem. Soc. Rev..

[19] Nam JM (2016). Plasmonic nanogap-enhanced Raman scattering with nanoparticles. Acc. Chem. Res..

[20] Lai HS (2020). Metal–organic frameworks: opportunities and challenges for surface-enhanced Raman scattering – a review. J. Mater. Chem. C..

[21] Li JF (2017). Core-shell nanoparticle-enhanced Raman spectroscopy. Chem. Rev..

[22] Zhang H (2020). Core-shell nanostructure-enhanced Raman spectroscopy for surface catalysis. Acc. Chem. Res..

[23] Jiang D (2014). Ag films annealed in a nanoscale limited area for surface-enhanced Raman scattering detection. Nanotechnology.

[24] Oh YJ (2016). Engineering hot spots on plasmonic nanopillar arrays for SERS: a review. BioChip J..

[25] Langer J (2020). Present and future of surface-enhanced Raman scattering. ACS Nano.

[26] Pérez-Jiménez AI (2020). Surface-enhanced Raman spectroscopy: benefits, trade-offs and future developments. Chem. Sci..

[27] Anderson MS (2000). Locally enhanced Raman spectroscopy with an atomic force microscope. Appl. Phys. Lett..

[28] Hayazawa N (2000). Metallized tip amplification of near-field Raman scattering. Opt. Commun..

[29] Stöckle RM (2000). Nanoscale chemical analysis by tip-enhanced Raman spectroscopy. Chem. Phys. Lett..

[30] Pettinger B (2000). Surface enhanced Raman spectroscopy: towards single molecule spectroscopy. Electrochemistry.

[31] Li JF (2010). Shell-isolated nanoparticle-enhanced Raman spectroscopy. Nature.

[32] Murphy DV (1982). Surface-enhanced hyper-raman scattering from SO_3_^2−^ adsorbed on Ag powder. Chem. Phys. Lett..

[33] Frontiera RR (2011). Surface-enhanced femtosecond stimulated Raman spectroscopy. J. Phys. Chem. Lett..

[34] Osawa, M. Surface-enhanced infrared absorption. in *Near-Field Optics and Surface Plasmon Polaritons* (ed Kawata, S., Springer, Berlin, Heidelberg, 2001).

[35] Neubrech F (2017). Surface-enhanced infrared spectroscopy using resonant nanoantennas. Chem. Rev..

[36] García de Abajo FJ (2014). Graphene plasmonics: challenges and opportunities. ACS Photonics.

[37] Rodrigo D (2015). Mid-infrared plasmonic biosensing with graphene. Science.

[38] Zhong YJ (2015). Review of mid-infrared plasmonic materials. J. Nanophotonics.

[39] Autore M (2018). Boron nitride nanoresonators for phonon-enhanced molecular vibrational spectroscopy at the strong coupling limit. Light. Sci. Appl..

[40] Massey GA (1985). Subwavelength resolution far-infrared microscopy. Appl. Opt..

[41] Knoll B, Keilmann F (1999). Near-field probing of vibrational absorption for chemical microscopy. Nature.

[42] Galabov, B. S. D. T. Vibrational Spectra and Structure (eds Boris, S. G. & Todor, D., Elsevier, 1996).

[43] Kerker M, Wang DS, Chew H (1980). Surface enhanced Raman scattering (SERS) by molecules adsorbed at spherical particles: errata. Appl. Opt..

[44] Roberto R, Claro F (1993). Theory of surface enhanced Raman scattering in colloids. J. Chem. Phys..

[45] Gersten J, Nitzan A (1980). Electromagnetic theory of enhanced Raman scattering by molecules adsorbed on rough surfaces. J. Chem. Phys..

[46] Willets KA, Van Duyne RP (2007). Localized surface plasmon resonance spectroscopy and sensing. Annu. Rev. Phys. Chem..

[47] Murray WA, Barnes WL (2007). Plasmonic materials. Adv. Mater..

[48] Stiles PL (2008). Surface-enhanced Raman spectroscopy. Annu. Rev. Anal. Chem..

[49] Boltasseva A, Atwater HA (2011). Low-loss plasmonic metamaterials. Science.

[50] Stanley R (2012). Plasmonics in the mid-infrared. Nat. Photonics.

[51] Raether, H. Surface Plasmons on Smooth and Rough Surfaces and on Gratings. (Berlin Heidelberg: Springer-Verlag, 1988).

[52] Liu Y (2011). Localized and propagating surface plasmon co-enhanced Raman spectroscopy based on evanescent field excitation. Chem. Commun..

[53] You EM (2021). Attenuated total reflection-cascading nanostructure-enhanced Raman spectroscopy on flat surfaces: a nano-optical design. J. Raman Spectrosc..

[54] Zhong JH (2017). Probing the electronic and catalytic properties of a bimetallic surface with 3 nm resolution. Nat. Nanotechnol..

[55] Sonntag MD (2012). Single-molecule tip-enhanced Raman spectroscopy. J. Phys. Chem. C..

[56] Huck C (2014). Surface-enhanced infrared spectroscopy using nanometer-sized gaps. ACS Nano.

[57] Kim J (2021). Single-particle analysis on plasmonic nanogap systems for quantitative SERS. J. Raman Spectrosc..

[58] Lim DK (2010). Nanogap-engineerable Raman-active nanodumbbells for single-molecule detection. Nat. Mater..

[59] Wei H, Xu HX (2013). Hot spots in different metal nanostructures for plasmon-enhanced Raman spectroscopy. Nanoscale.

[60] Dodson S (2013). Optimizing electromagnetic hotspots in plasmonic bowtie nanoantennae. J. Phys. Chem. Lett..

[61] McMahon JM, Gray SK, Schatz GC (2011). Fundamental behavior of electric field enhancements in the gaps between closely spaced nanostructures. Phys. Rev. B.

[62] Dong L (2017). Nanogapped Au antennas for ultrasensitive surface-enhanced infrared absorption spectroscopy. Nano Lett..

[63] Alvarez-Puebla RA (2011). Gold nanorods 3D-supercrystals as surface enhanced Raman scattering spectroscopy substrates for the rapid detection of scrambled prions. Proc. Natl Acad. Sci. USA.

[64] Wang H, Kundu J, Halas NJ (2007). Plasmonic nanoshell arrays combine surface-enhanced vibrational spectroscopies on a single substrate. Angew. Chem. Int. Ed..

[65] Le F (2008). Metallic nanoparticle arrays: a common substrate for both surface-enhanced Raman scattering and surface-enhanced infrared absorption. ACS Nano.

[66] Lim DK (2011). Highly uniform and reproducible surface-enhanced Raman scattering from DNA-tailorable nanoparticles with 1-nm interior gap. Nat. Nanotechnol..

[67] Li JF (2011). Extraordinary enhancement of Raman scattering from pyridine on single crystal Au and Pt electrodes by shell-isolated Au nanoparticles. J. Am. Chem. Soc..

[68] Chen S (2016). How to light special hot spots in multiparticle-film configurations. ACS Nano.

[69] Li JF (2013). In situ SHINERS at electrochemical single-crystal electrode/electrolyte interfaces: tuning preparation strategies and selected applications. ACS Nano.

[70] Li CY (2015). In situ monitoring of electrooxidation processes at gold single crystal surfaces using shell-isolated nanoparticle-enhanced Raman spectroscopy. J. Am. Chem. Soc..

[71] Dong JC (2016). Shell-isolated nanoparticle-enhanced Raman spectroscopy at single-crystal electrode surfaces. Adv. Optical Mater..

[72] Bodappa N (2019). Early stages of electrochemical oxidation of Cu(111) and polycrystalline Cu surfaces revealed by in situ Raman spectroscopy. J. Am. Chem. Soc..

[73] Dong JC (2020). Direct in situ Raman spectroscopic evidence of oxygen reduction reaction intermediates at high-index Pt(*hkl*) surfaces. J. Am. Chem. Soc..

[74] Li CY (2019). In situ probing electrified interfacial water structures at atomically flat surfaces. Nat. Mater..

[75] Su M (2020). In situ Raman study of CO electrooxidation on Pt(*hkl*) single-crystal surfaces in acidic solution. Angew. Chem. Int. Ed..

[76] Dong JC (2019). In situ Raman spectroscopic evidence for oxygen reduction reaction intermediates at platinum single-crystal surfaces. Nat. Energy.

[77] Zhang R (2013). Chemical mapping of a single molecule by plasmon-enhanced Raman scattering. Nature.

[78] Lu F, Jin MZ, Belkin MA (2014). Tip-enhanced infrared nanospectroscopy via molecular expansion force detection. Nat. Photonics.

[79] Benz F (2016). Single-molecule optomechanics in “picocavities”. Science.

[80] Lee J (2019). Visualizing vibrational normal modes of a single molecule with atomically confined light. Nature.

[81] Mahapatra S (2020). Tip-enhanced Raman spectroscopy: chemical analysis with nanoscale to angstrom scale resolution. J. Chem. Phys..

[82] Pozzi EA (2017). Ultrahigh-vacuum Tip-enhanced Raman spectroscopy. Chem. Rev..

[83] Chiang N (2017). Probing intermolecular vibrational symmetry breaking in self-assembled monolayers with ultrahigh vacuum tip-enhanced Raman spectroscopy. J. Am. Chem. Soc..

[84] Chiang N (2016). Conformational contrast of surface-mediated molecular switches yields angstrom-scale spatial resolution in ultrahigh vacuum tip-enhanced Raman spectroscopy. Nano Lett..

[85] Jiang N (2016). Nanoscale chemical imaging of a dynamic molecular phase boundary with ultrahigh vacuum tip-enhanced Raman spectroscopy. Nano Lett..

[86] Zhu WQ, Crozier KB (2014). Quantum mechanical limit to plasmonic enhancement as observed by surface-enhanced Raman scattering. Nat. Commun..

[87] Savage KJ (2012). Revealing the quantum regime in tunnelling plasmonics. Nature.

[88] Ding SY (2017). Further expanding versatility of surface-enhanced Raman spectroscopy: from non-traditional SERS-active to SERS-inactive substrates and single shell-isolated nanoparticle. Faraday Discuss..

[89] Mubeen S (2012). Plasmonic properties of gold nanoparticles separated from a gold mirror by an ultrathin oxide. Nano Lett..

[90] Huang C (2008). Gain, detuning, and radiation patterns of nanoparticle optical antennas. Phys. Rev. B.

[91] Shegai T (2011). Angular distribution of surface-enhanced Raman scattering from individual au nanoparticle aggregates. ACS Nano.

[92] Fu YH (2013). Directional visible light scattering by silicon nanoparticles. Nat. Commun..

[93] Li NN (2021). Directional control of light with nanoantennas. Adv. Optical Mater..

[94] Shegai T (2011). A bimetallic nanoantenna for directional colour routing. Nat. Commun..

[95] Wang HL (2017). Integrated plasmon-enhanced Raman scattering (*i*PERS) spectroscopy. Sci. Rep..

[96] Meyer SA, LeRu EC, Etchegoin PG (2011). Combining surface plasmon resonance (SPR) spectroscopy with surface-enhanced Raman scattering (SERS). Anal. Chem..

[97] Meyer SA (2012). Combined SPR and SERS microscopy in the Kretschmann configuration. J. Phys. Chem. A.

[98] McKee KJ, Smith EA (2010). Development of a scanning angle total internal reflection Raman spectrometer. Rev. Sci. Instrum..

[99] Nyamekye CKA (2018). Combined measurement of directional Raman scattering and surface-plasmon-polariton cone from adsorbates on smooth planar gold surfaces. Analyst.

[100] Liu Y (2010). Note: simultaneous measurement of surface plasmon resonance and surface-enhanced Raman scattering. Rev. Sci. Instrum..

[101] Xue XK (2008). Practically modified attenuated total reflection surface-enhanced IR absorption spectroscopy for high-quality frequency-extended detection of surface species at electrodes. Anal. Chem..

[102] Xu, W. Q. *et al*. A device for integrated plasmon-enhanced Raman with adjustable excitation angle (in Chinese), China Patent CN206270249U (2017).

[103] Zhao, K. H. & Zhong, X. H. Optics. Ch 1. (Peking University Press, China, 1984).

[104] Kamp M (2020). Cascaded nanooptics to probe microsecond atomic-scale phenomena. Proc. Natl Acad. Sci. USA.

[105] Zeng ZC (2015). Electrochemical tip-enhanced Raman spectroscopy. J. Am. Chem. Soc..

[106] Huang SC (2020). Probing nanoscale spatial distribution of plasmonically excited hot carriers. Nat. Commun..

[107] Kim S (2019). High external-efficiency nanofocusing for lens-free near-field optical nanoscopy. Nat. Photonics.

[108] Zhang Y (2019). Visually constructing the chemical structure of a single molecule by scanning Raman picoscopy. Natl Sci. Rev..

[109] Wang ZB (2011). Optical virtual imaging at 50 nm lateral resolution with a white-light nanoscope. Nat. Commun..

[110] Jin MZ, Lu F, Belkin MA (2017). High-sensitivity infrared vibrational nanospectroscopy in water. Light.: Sci. Appl..

[111] Wang HM (2020). Probing mid-infrared phonon polaritons in the aqueous Phase. Nano Lett..

